# Psychometric Properties of the Revised Self-Efficacy for Diabetes Self-Management Scale among Spanish Children and Adolescents with Type 1 Diabetes †

**DOI:** 10.3390/children11060662

**Published:** 2024-05-29

**Authors:** Joaquín Villaécija, Bárbara Luque, Esther Cuadrado, Sebastián Vivas, Carmen Tabernero

**Affiliations:** 1 Department of Psychology, University of Cordoba, 14071 Cordoba, Spain; jvillaecija2@uco.es (J.V.); svivas@uco.es (S.V.); 2 Maimonides Biomedical Research Institute of Cordoba (IMIBIC), 14004 Cordoba, Spain; 3 Reina Sofia University Hospital (HURS), 14004 Cordoba, Spain; 4 Department of Social Psychology and Anthropology, University of Salamanca, 37005 Salamanca, Spain; carmen.tabernero@usal.es; 5 Instituto de Neurociencias de Castilla y León (INCYL), University of Salamanca, 37005 Salamanca, Spain

**Keywords:** self-efficacy, self-management, type 1 diabetes, longitudinal study, psychometric study, children, adolescents

## Abstract

A longitudinal design was used to examine the psychometric properties of the Self-Efficacy for Diabetes Self-Management (SEDM) for children and adolescents with a diagnosis of type 1 diabetes (T1D). The SEDM was adapted to Spanish and the best factorial solution was selected to test the invariance of the measures of age and gender. Individuals between the ages of 10 and 19 years old with a diagnosis of T1D completed a self-reported questionnaire (167 at Time 1 [mean age = 14.49, SD = 2.76; 56.9% boys] and 122 at Time 2 [mean age = 14.77, SD = 2.58; 56.6% boys]). Two unifactorial solutions were tested. The psychometric properties of the scale were validated. The proposed validation obtained excellent reliability indices (χ^2^ (26) = 25.59, *p* > 0.49, RMSEA = 0.00, 95% CI [0.00, 0.07], CFI = 1.00, GFI = 0.96, AGFI = 0.92, TLI = 1.00, and CMIN = 0.98), and it appeared to be invariant for gender and for age groups. The Cronbach’s α was 0.85. The test–retest reliability was high (r = 0.69 [*p* < 0.001]). Convergent, discriminant, and external validity were proven. The nine-item SEDM is a brief measure with satisfactory structural validity. From our knowledge, this study provides the first reliable tool to assess self-efficacy in the management of T1D for Spanish children and adolescents.

## 1. Introduction

Type 1 diabetes (T1D) is caused by the destruction of the β cells that produce insulin in the pancreas [1]. T1D requires both the self-management of insulin therapy throughout life and the monitoring of blood glucose [2]. The treatment of T1D requires the daily administration of insulin. Insulin requirements are adjusted according to glycemic control, values that vary according to food intake, physical activity, menstrual cycle, or psychosocial variables [3,4,5]. Mostly, the loss of these cells primarily occurs because of autoimmunity related to the disease, while, in a small group, the causes are unknown (called idiopathic T1D), with a strong genetic component. In any case, it is one of the most prevalent endocrine and metabolic diseases in childhood [6]. These individuals are at an increased risk for other autoimmune diseases and psychosocial problems [7]. It is estimated that 18% of those affected are under 20 years of age and that the prevalence could double by 2040 [8]. Scientific advances in recent years show that environmental factors may also be behind this increased prevalence, and there has even been talk of population screening to delay or prevent the onset of T1D [9,10,11].

T1D is a chronic condition with an early debut, and children with T1D live many years with this pathology. Therefore, the behavioural component is crucial for the effective self-regulation of diabetes. To understand human behaviour, the influence of psychosocial variables must not be overlooked, and, for this purpose, it is impossible not to resort to Bandura’s Social Cognitive Theory (SCT). SCT explains that individuals themselves are agents of change capable of both influencing and being influenced by the environment [12]. Self-efficacy takes on a special relevance in behaviour modification in childhood and adolescence [13]. Self-efficacy is defined as a person’s self-confidence in relation to the achievement of a certain goal [14] and is shown to be an essential variable in numerous studies with children and adolescents with T1D for a variety of reasons. Among them, a notable finding is the mediating role of self-efficacy between perceived stress and the self-management of the T1D [15]. 

In addition to the construct of self-efficacy itself, its relationship with other psychosocial variables justifies the influence it may have on diabetes management. A positive relationship was found between self-efficacy and the understanding of diabetes along with its treatment [16], which improves the quality of life in the transition to adulthood [17] and may even improve glycemic control [18]. Not least, safe environments and, above all, quality social support have proven to be very important in increasing confidence in disease management [19]. Parental communication of autonomy support is associated with better treatment compliance in adolescents with T1D [20].

The theory itself takes the causal structure pathway to argue for a belief in self-efficacy in the regulation of behaviour associated with one’s own health [21]. Hand in hand with self-efficacy, there are outcome expectations (positive and negative), an anticipation of the consequence that may have a reinforcing effect on the behaviour [11]. This premise has already been demonstrated in clinical settings: the patient’s own goal-setting (outcome expectations) makes him or her more likely to follow the clinician’s recommendations [22]. Likewise, positive emotional health would be associated with a more efficient management of diabetes [23]. Furthermore, the better regulation of negative emotions in adolescents makes it easier for them to cope with their goals [24]. In any case, it is recommended that children with chronic diseases work on self-management and self-efficacy skills to improve their health [25]. Diabetes management was one of the most frequently mentioned concerns in the early adolescence of patients with T1D [26].

This study aims to develop a short, psychometrically sound instrument to measure self-efficacy for managing T1D in a Spanish child and adolescent population with T1D. To this end, the Self-Efficacy for Diabetes Self-Management (SEDM) scale was validated [27]. Its psychometric properties were good, showing reliability and predictive validity. Two models were compared. Model 0 (M0) applies a confirmatory factor analysis (CFA) and respects the ten-item unifactorial solution presented in the original study. Model 1 (M1) verifies step by step the psychometric properties of the instrument before applying the CFA (which was finally carried out with a solution that is also unifactorial, but, in this case, with nine items). The CFA was not applied in the original study but was conducted using a longitudinal sample of infant–juvenile individuals with T1D (two times). The analysis included test–retest reliability, measurement invariance, and the assessment of convergent, discriminant, and external validity.

## 2. Materials and Methods

### 2.1. Design

A descriptive instrumental quantitative study was designed using a randomised longitudinal survey. The longitudinal design was used to verify the validity of the results over time.

### 2.2. Procedure

First, the SEDM items were double-back-translated into Spanish by an expert panel. Following a pilot survey, the reliability of the questionnaire was tested in an age-matched target population. Then, once the research design was completed and approval was received from the Cordoba Research Ethics Committee (date and version of the protocol: 1–06/01/2020; committee reference: 5166; report no. 327; approved on 28 September 2021), the fieldwork was conducted at the endocrinology units during the patients’ regular check-up appointments with their physicians. Inclusion criteria included: children aged between 10 and 19 years old with a diagnosis of T1D who attended the Endocrinology or Paediatrics Unit of the Reina Sofía University Hospital in Cordoba (Spain). In addition, children had to be native speakers or fluent in Spanish. After explaining the research objective and the participation process to the families, the parents of minors and young participants of legal age signed a consent form. Each child completed a self-report questionnaire via an online design at Questback Unipark (Cologne, Germany) with tablets supplied by our research team. A research team member was always present during data collection in case the younger patients needed help. After completing the questionnaire (approximately 25 min), the patient’s medical report was provided by the medical team. The interval between T1 (Time 1) and T2 (Time 2) differed according to the age of the individual: 3 months for those under 14 years of age and 6 months for older individuals. The difference in follow-up time for the ages described was due to the organisation of the healthcare system in Spain. The follow for T1D in the paediatric age group is conducted approximately every 3 months until the age of 14. At this point, adolescents transition to adult endocrinology care, and their follow-up occurs every 6 months.

### 2.3. Measures

#### 2.3.1. Sociodemographic and Clinical Data

Each child was asked their age and sex, while data about the disease onset, the disease duration (in years), and blood glucose level (HbA1c in mg/dL) were collected from the medical report if the data were available on the day the survey was conducted.

#### 2.3.2. Self-Efficacy for Diabetes Self-Management

The short version of the SEDM was selected [27]. Individuals used a five-point Likert scale (1 = nothing sure to 5 = absolutely sure) to score the likelihood of behaviour in relation to the management of T1D. After translation into Spanish, two unifactorial models were tested. Cronbach’s α in the original scale was 0.90, while, in our sample, it was 0.83 for M0 and 0.85 for M1.

#### 2.3.3. Positive Outcome Expectations of Diabetes Self-Management

The short version of Positive Outcome Expectations of Diabetes Self-Management (OEDM-P) was used to measure positive expectations regarding patients’ diabetes management, one of the factors resulting from the scale of the Outcome Expectations of Diabetes Self-Management (OEDM) [27]. The instrument required adaptation prior to translation. The Spanish state covers the health needs of this population without financial charge, so two items related to the expectations of cost savings were eliminated. Individuals answered on a five-point Likert scale (1 = nothing sure to 5 = absolutely sure). Cronbach’s α in the original study was 0.84, and it was 0.78 in our study.

#### 2.3.4. Perceived Social Support

To measure perceived social support, the Spanish version of the Multidimensional Scale of Perceived Social Support (MSPSS) was used [28]. Individuals scored 12 items on a five-point Likert scale (1 = strongly disagree to 5 = in full agreement) according to the degree of conformity with the statement. This scale consisted of three factors of four items each: Family, Friends, and Significant other. The original version presented a Cronbach’s α of 0.87, 0.85, and 0.91, respectively, while, in the validation used, it was 0.85, 0.89, and 0.79, respectively. For our sample, Cronbach’s α was 0.90 (Family), 0.89 (Friends), and 0.84 (Significant other).

#### 2.3.5. Positive Affect and Negative Affect

A short Spanish version of the Positive Affect and Negative Affect Scale (PANAS) was used, which was divided into two factors: positive affect (PA) and negative affect (NA) [29]. Both the original and the validated versions showed good internal consistency: α = 0.86 and α = 0.77, respectively, for PA; and α = 0.82 and α = 0.79, for NA. Cronbach’s α was 0.93 (PA) and 0.86 (NA) in our sample. Individuals answered on a five-point Likert scale (1 = nothing to 5 = totally).

### 2.4. Statistical Analyses

A descriptive analysis of the sociodemographic data of the sample and the calculation of Cronbach’s α of the instruments used was the beginning of the statistical analyses performed. For consistent reliability of the instrument, Cronbach’s α must be greater than 0.70 [30]. Subsequently, we worked with the Spanish validation of SEDM for children and youth populations. We tested the one-factor structure of the original scale (through an exploratory factor analysis [EFA]) with the patient sample at T1 in the first phase. From here, the comparison between the two models emerged. To interpret the adjustment indices in the CFA, the recommendations from Schermelleh-Engel et al. (2003) were used [31]. The values considered were chi-square (χ^2^), goodness-of-fit index (GFI), adjusted goodness-of-fit index (AGFI), the comparative fit index (CFI), the root-mean-square error of approximation (RMSEA), and the Tucker–Lewis index (TLI). The model was tested again with the patient sample at T2, and the test–retest reliability of the validated instrument was assessed using Spearman’s correlation. The measurement invariance was tested for gender and age groups. Lastly, the validity of the instrument was examined through convergent, discriminant, and external validity analyses by correlating it (also using Spearman’s correlation coefficient) and computing a series of linear regression with different variables in the study that have theoretical consistency. The significance or non-significance of the β value (*p* < 0.05) will determine the predictive effect of that variable on the dependent variable. Statistical Package for Social Sciences (SPSS.25) and Amos.24 were the software used.

## 3. Results

### 3.1. Sample Demographics

Of all the families that met the inclusion criteria and were asked to participate in the study, 74.22% agreed to participate, 167 consented at T1 (mean age = 14.49, SD = 2.76; 56.9% boys), and 122 consented at T2 (mean age = 14.77, SD = 2.58; 56.6% boys). The descriptive sociodemographic and clinical aspects of the sample are shown in Table 1.

### 3.2. Correlation between Items

Correlation analyses were performed. All items except 3 (“Exercise even when I don’t feel like it”) showed an adequate correlation (r > 0.20) with half or more of them. Following this, item 3 was removed in M1 due to its poor correlation with the other items.

### 3.3. Reliability Analyses

M0 showed a reliability level of 0.83. M1 presented a Cronbach’s α of 0.85. The analysis with M0 also reported an improvement in the coefficient if item 3 were removed (giving rise, if it were implemented, to the same solution executed in M1).

### 3.4. Exploratory Factor Analysis

For nine items of the SEDM (M1), the Kaiser–Meyer–Olkin (KMO) index (0.88) and Bartlett’s sphericity test (BTS [χ^2^ = 487.82; df = 36; *p* < 0.001]) supported the use of the EFA. The EFA was conducted with Varimax rotation, and the results showed a unifactorial solution. This structure explained 45.90% of the variance. On the other hand, the unifactorial solution proposed by the authors (M0) who originally designed it explained 42.53% of the variance. Table 2 shows the standardised SEDM regression coefficients for this unifactorial solution in M0 and M1. As can be observed, and as anticipated in the correlation analysis between items, item 3 (maintained in the M0 as in the original scale) showed a factor load of less than 0.40, suggesting its unimportance.

**Figure 1:**
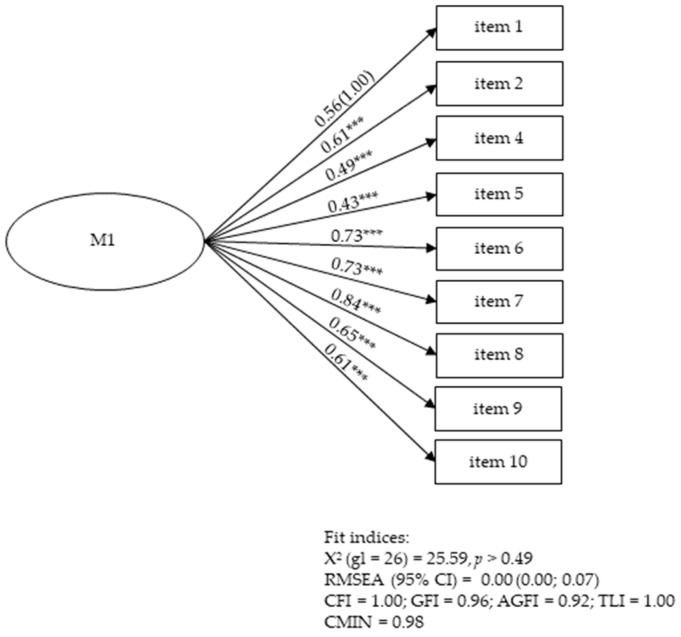
CFA standardised estimates of SEDM at T2. Note: *** *p* < 0.001.

Two models were examined with the CFA at T2. The M0 revealed good scores: χ^2^ (34) = 41.15, *p* > 0.19, RMSEA = 0.04, 95% CI [0.00, 0.08], CFI = 0.98, GFI = 0.94, AGFI = 0.90, TLI = 0.98, and CMIN = 1.21. Subsequently, M1 was tested, which consisted of a nine-item unifactorial version where item 3 was removed. As observed (Figure 1), M1 exhibited improved (excellent) fit indices compared M0.

### 3.5. Test–Retest Reliability

Test–retest reliability was tested using data from both time points. The correlation between T1 and T2 for M0 was r = 0.69 (*p* < 0.001), while, for M1, the correlation was r = 0.69 (*p* < 0.001). The result was high, which means that the scale has adequate test–retest reliability.

### 3.6. Multigroup Analysis: Gender and Age Invariance

Table 3 shows the measurement invariance. To test the invariance, two variables were selected: gender and age (establishing two age groups: 10–13 years old and older than 14 years old). The model was invariant for gender and was valid for both males and females for both age groups.

### 3.7. Convergent, Discriminant, and External Validity Analyses

The analysis of convergent, discriminant, and external validity confirmed that the SEDM showed expected relationships with other variables included in the study. Firstly, the SEDM exhibited a positive correlation with OEDM-P (*p* < 0.001). Moreover, the three dimensions of the MSPSS dimensions (Family, Friends, and Significant other) demonstrated a strong positive correlation with the SEDM (*p* < 0.001). In relation to affection, the SEDM correlated positively with positive affect (*p* < 0.001), whereas, as expected, it correlated negatively with negative affect (*p* < 0.001). No correlations were shown between the SEDM and clinical variables (neither with HbA1c nor with disease duration), although it correlated marginally with HbA1c (*p* < 0.10).

A series of linear regressions were also performed at both times with those variables in which self-efficacy for diabetes management could theoretically act as a predictor variable. The SEDM acted as a predictor variable for OEDM-P, positive affect and negative affect at both times, and for HbA1c at T1, as shown in Table 4.

The regression analysis shows how self-efficacy for managing diabetes in children and adolescents not only is related to psychological variables, but also influences biomedical variables, such as HbA1.

## 4. Discussion

The main aim of this study was to establish a short psychometrically sound instrument for assessing self-efficacy in the management of T1D among Spanish children and adolescents. For this purpose, it was decided that we should validate the SEDM scale in this population [27]. To the best of our knowledge, this is the first time that this questionnaire has been validated in Spanish, and it is the only known tool to measure self-efficacy for the management of T1D in children and adolescents in this language. 

The CFA results indicated that the one-factor model based on the proposed translation of the original 10-item version (M0) demonstrated good-fit indices. However, the nine-item version (M1), which involved removing item 3 (“Exercise even when I don’t feel like it”), exhibited even better-fit indices, reaching an excellent level. It is worth considering that the negative wording of item 3 may have led to potential confusion or misinterpretation among the surveyed individuals, resulting in a considerably low factor loading. This item was not discarded in the initial pilot test because its removal did not improve the reliability of the instrument; hence, the expert panel decided to keep it. Therefore, we proceeded with the nine-item version after the CFA analysis. This unifactorial solution remained consistent during the interval from one medical follow-up to the next, between three months and six months, with excellent fit rates. The scale showed adequate internal consistency and high test–retest correlation coefficients, demonstrating its reliability. In addition, measurement invariance was assessed for gender and age groups, resulting in invariance in both cases. Previous studies have suggested that gender can influence how young individuals manage diabetes and how parents help, with differences observed between sons and daughters [32]. The gender invariance of the instrument is highly significant, as it provides a valid measure for both boys and girls, especially considering that T1D is the only autoimmune disease with a higher prevalence among males after puberty [33]. In turn, the instrument’s utility was demonstrated across the two defined age groups, indicating that younger individuals effectively comprehended the translated items. This separation of age groups was made because children up to 14 years of age in Spain are treated in paediatrics, while older patients are treated in adult units. 

Convergent, discriminant, and external validity were evaluated by examining the relationships with other psychosocial variables. Consistent with Bandura’s SCT [14], self-efficacy for diabetes management not only exhibited positive correlations with positive outcome expectations related to T1D but was also shown to be a predictor. A recent review considers that cognitive behavioural therapy in adolescents with T1D may be effective [34]. On the other hand, and in line with previous studies, positive correlations were shown between self-efficacy for diabetes control and perceived social support. In this sense, the correlation between perceived social support and self-care behaviours in these individuals, especially when this support comes directly from the family environment, has already been discussed [19,35]. Undoubtedly, safe and trusting environments seem to facilitate the adoption of healthy behaviours and help people remain emotionally stable. The SEDM also acted as a predictor of emotional well-being through positive affect, negative affect, and HbA1c. This is in line with other studies which place self-efficacy in healthy adolescents as a predictor variable of subjective well-being [24].

The management of T1D is complex because it requires adjusting the daily administration of insulin according to other variables such as food intake, the level of physical activity performed, or stress compared to peers in adolescence [36]. Therefore, children and adolescents with T1D need self-care for disease control. In adolescence, this control is complicated by highly variable insulin requirements, and diet and activity are often unpredictable during this stage, and the assumption of adult roles. The validity of this instrument, not only for children, but also for adolescents and young adults, is an important step in measuring their belief in their own ability to manage T1D, at a time of transition when multiple factors, both biological and psychosocial, determine an increase in risk factors for poorer adjustment to this condition [37].

This study has some limitations that should be mentioned. The sample size and the methodological aspects were in line with similar studies on adaptations and validations of psychological scales. However, an improvement would be to have a multicenter design that includes a population of children and adolescents with T1D from different geographical locations. On the other hand, although the study included psychosocial variables (positive expectations regarding diabetes management, perceived social support, and positive and negative affect), it could include other dispositional or personality variables. Similarly, other biomedical variables, such as time in range or time in hyperglycemia, could be incorporated in future studies. It would also be interesting to add some psychosocial variables of the parents, especially related to the management of T1D in their children, similar to those assessed by the study of the original scale [27].

The absence of instruments to measure self-efficacy for the management of T1D in the Spanish population of children and adolescents with T1D makes this study a relevant contribution to the scientific field with imminent clinical usability. Furthermore, the validation was carried out with a methodological design that had many strengths: (1) a longitudinal design was used to ensure the maintenance of the measurements; (2) the participation of a large clinical sample of both paediatric and adolescent minors was achieved; (3) an initial EFA confirmed the one-dimensionality of the original scale, and an executed CFA proved that the Spanish validation was valid with excellent fit rates; (4) the internal consistency and an adequate reliability test–retest were tested; (5) the measurement invariance of the instrument was tested for gender and age groups; and (6) convergent, discriminant, and external validations were verified with correlations and a series of linear regressions in line with the scientific literature.

## 5. Conclusions

The simple composition of nine items in a single-factor solution provides an easy-to-use tool to assess self-efficacy in the management of T1D in Spanish children and adolescents. This tool can be used by different professionals involved in the education, treatment, and care of this population. The Spanish SEDM scale could help to predict behaviours in relation to diabetes management and, as has been shown, positive expectations in relation to that management, as well as their emotional well-being. In addition, this tool may also be very useful for testing the efficiency of psychoeducational interventions based on self-efficacy. The Spanish SEDM scale can improve beliefs about the management of T1D in children and adolescents and offer a more comprehensive approach for their treatment, which could lead to significant advances in actual control soon.

## Figures and Tables

**Table 1:** Demographic and clinical data.

Demographic and Clinical Data	T1	T1_boys_	T1_girls_	T2	T2_boys_	T2_girls_
*N* (%)	167 *	95 (56.9)	71 (42.5)	122	69 (56.6)	53 (43.4)
Age (*M*, *SD*)	14.49 (2.76)	14.62 (2.66)	14.28 (2.89)	14.77 (2.58)	14.65 (2.29)	14.92 (2.93)
Age at diagnosis (*M*, *SD*), years	8.80 (4.28)	9.28 (4.39)	8.21 (4.08)	8.70 (4.28)	9.1 (4.22)	8.17 (4.34)
Disease duration (*M*, *SD*), years	5.68 (4.24)	5.34 (4.09)	6.06 (4.39)	5.57 (4.17)	5.01 (3.99)	6.28 (4.31)
Regimen						
- Basal-bolus injections (*n*, %)	153 (91.6)	88 (92.6)	64 (90.1)	111 (91)	65 (94.2)	46 (86.8)
- Insulin infusion pump (*n*, %)	14 (8.4)	7 (7.4)	7 (9.9)	11 (9)	4 (5.8)	7 (13.2)
HbA1c (mg/dL)	7.55 (1.51)	7.58 (1.71)	7.51 (1.15)	7.59 (1.54)	7.77 (1.83)	7.34 (1)

* One patient identified as non-binary sex in T1 (0.6%). Note: HbA1c was analysed when was available in the medical report (*n* = 104 in T1, *n* = 83 in T2).

**Table 2:** Standardised regression coefficients for self-efficacy for diabetes self-management.

Item No.	Item Text	Original Scale	Spanish Validation of the Original Version (M0)	Alternative Spanish Validation (M1)
1	Adjust your insulin correctly when you eat more or less than usual.	0.53	0.62	0.63
2	Choose healthful foods when you go out to eat.	0.63	0.64	0.63
3	Exercise even when you don’t really feel like it.	0.53	0.39	-
4	Adjust your insulin or food accurately based on how much exercise you get.	0.65	0.73	0.62
5	Talk to your doctor or nurse about any problems you’re having with taking care of your diabetes.	0.78	0.53	0.54
6	Do your blood sugar checks even when you are really busy.	0.83	0.76	0.77
7	Manage your diabetes the way your healthcare team wants you to.	0.85	0.75	0.75
8	Manage your diabetes even when you feel overwhelmed.	0.82	0.80	0.81
9	Find ways to deal with feeling frustrated about your diabetes.	0.77	0.62	0.62
10	Identify things that could get in the way of managing your diabetes.	0.78	0.68	0.69

**Table 3:** Tests of invariance for gender and age groups.

Test of Invariance	χ^2^	df	CFI	Δχ^2^	Δdf	*p*
Configural_gender_	59.71	52	0.98			
Metric_gender_	69.03	60	0.98	9.32	8.00	0.32
Scalar_gender_	69.23	61	0.98	0.20	1.00	0.65
Residual_gender_	74.04	71	0.98	4.81	10.00	0.90
Configural_age_	49.99	52	1.00			
Metric_age_	57.55	60	1.00	7.56	8.00	0.48
Scalar_age_	59.71	61	1.00	2.16	1.00	0.14
Residual_age_	62.80	71	1.00	3.09	10.00	0.75

**Table 4:** Regression analyses of the SEDM and other variables.

	Time 1			Time 2		
	β	F(1, 165) ^a^	R^2^	β	F(1, 120) ^b^	R^2^
OEDM-P		14.29 ***	0.08		14.27 ***	0.10
SEDM	0.28 ***			0.33 ***		
PA		40.97 ***	0.19		29.69 ***	0.20
SEDM	0.45 ***			0.46 ***		
NA		24.23 ***	0.13		11.05 ***	0.08
SEDM	−0.36 ***			−0.29 **		
HbA1c		4.00 *	0.04		3.43 ^#^	0.04
SEDM	−0.45 *			−0.20 ^#^		

Note: β: Standardised estimates; OEDM-P: Positive Outcome Expectations of Diabetes Self-Management; PA: Positive affect; NA: Negative affect. ^#^ *p* < 0.10; * *p* < 0.05; ** *p* < 0.01; *** *p* < 0.001. ^a^ F(1, 102) for HbA1c at T1. ^b^ F(1, 81) for HbA1c at T2.

## Data Availability

Data are contained within the article.
